# Impact of Emerging Transport Technologies on Freight Economic and Environmental Performance: A System Dynamics View

**DOI:** 10.3390/ijerph192215077

**Published:** 2022-11-16

**Authors:** Taolei Guo, Junjie Chen, Pei Liu

**Affiliations:** Department of Logistics Management, Business School, Shandong University, Weihai 264209, China

**Keywords:** freight transport, road transport, technology, greenhouse gas emission, sustainability

## Abstract

Road freight transport promotes economic development while impeding the future of green development due to excessive fossil fuel use. Road freight enterprises need to adapt to stricter environmental regulations while maintaining a reasonable level of profit. However, this is not easy in a growing economy such as China’s, whose domestic freight demand is increasing rapidly with economic growth. The development of emerging transport technologies (ETTs) creates great potential for reducing the negative environmental impact of road freight transport. This study considers five candidate ETTs: eco-driving, fleet platooning, vehicle utilization, optimized vehicle design, and renewable energy trucks. A system dynamics analytical framework is established to explore the long-term impact of ETTs on road profit and greenhouse gas (GHG) emissions under the uncertainty of macroeconomic development. Road freight enterprises affiliated with the Qingdao port in China are taken as a case study. The economic and environmental impact of their adoption of ETTs is projected from 2020 to 2035. The results show that the economic growth in the port hinterland leads to an increase in road freight volume and profit, but it also yields a greater amount of GHG emissions from road transport. All of the candidate ETTs exhibit a positive effect on reducing GHG emissions from road transport, but they also cause profit losses due to a high application cost, even though they reduce transport operating costs by fuel savings. The results of the Sobol sensitivity analysis show that GHG reductions are sensitive to the adoption of ETTs. Thus, a carbon-based compensation mechanism is introduced. With this mechanism, road freight enterprises should prioritize vehicle utilization, optimized vehicle design, and eco-driving in their adoption of ETTs for more sustainable development. The results provide systems-based insights into ETT deployment decisions for road freight companies.

## 1. Introduction

Freight transport connects the supply and demand for materials and finished products in the production process. It also provides opportunities for creating jobs and economic growth during the construction of transport infrastructures. Thus, freight transport plays an important role in supporting the regional economic development and economic performance of freight enterprises [1]. However, the excessive use of fossil fuels in freight transport activities also exerts negative impacts on the natural environment and energy safety. In the context of economic growth (particularly in China), the surge in freight demand will place pressure on transport infrastructure and spur the deterioration of the regional environment [2]. The environmental issues related to freight transport in China are considered. The energy consumption and carbon emissions in the freight sector in China are experiencing more rapid growth than those in developed countries and emerging economies [3].

In such circumstances, it is important to limit the negative impact of freight transport on the environment while ensuring the support of freight transport for economic development. In 2016, the State Council of China released *The Outline of Building a Powerful Country in Transportation*, which highlights the goal of establishing high-quality and energy-efficient regional transport systems. This goal involves efforts at both the policy level and the technology level. Policies facilitate the design and construction of infrastructure networks and tax and fee reductions to increase the efficiency of the entire freight market [4]. In academia, there are many analyses and interpretations of the impacts of policies [5]. Technologies are more relevant to the behavior of freight enterprises, that is, the adoption of emerging transport technologies (ETTs), such as eco-driving [6], fleet platooning [7], vehicle utilization [8], optimized vehicle design [9], and renewable energy trucks [10]. These ETTs are especially important to the sustainable development of road freight enterprises, whose environmental performance is lower than that of railway and waterway carriers. However, studies regarding the impact of these ETTs on the profitability and environmental performance of road freight enterprises are still limited.

In addition to the impact of ETTs, the profitability and environmental performance of road freight enterprises are influenced by economic circumstances, such as economic structural changes [1], shippers’ preferences for transportation modes (Xie and Lin, 2017), and competition from other transport modes [11]. Furthermore, the operation of freight enterprises interacts with the freight market [12]. Dynamic changes in the freight market complicate the decision-making of freight enterprises. This situation is particularly true for road freight enterprises affiliated with ports, which are a strategic endowment that connects global and local economies. In this study, we focus on the road freight enterprises serving the gathering and distribution of freight for the Qingdao port in China. These companies attract our attention because they need to adapt to the new regional economic development condition, which is induced by a major initiative called *The Kinetic Conversion from Old to New Industries* implemented in Shandong Province (the main economic hinterland of Qingdao port) [13].

The aim of this study is to explore the long-term impact of ETTs on complying with the targets of improving operational efficiency and environmental protection while considering the uncertainty in macroeconomic development. Specifically, this study attempts to answer the following two questions.

- First, what are the potential impacts of the economic transformation in port hinterlands on the operation of road freight enterprises? *The Kinetic Conversion from Old to New Industries* initiative aims to reduce traditional coal- and metal-related industries while stimulating new high-value-added industries in Shandong Province. This transition in the structure of the hinterland economy will influence the throughput of Qingdao port and further influence the freight demand of road freight enterprises.
- Second, how does the adoption of ETTs affect the operational efficiency, profitability, and environmental performance (primarily in terms of greenhouse gas (GHG) emissions) of road freight enterprises? The adoption of ETTs changes the structure of transport costs and further affects road freight companies’ competitive position in the freight market. Thus, its systematic influence on the profitability and environmental performance of road freight companies deserves dedicated investigation.

This study investigates the above two issues in a holistic manner by developing a system dynamics analytical framework. This research makes two main contributions. First, the proposed system dynamics framework integrates the econometric method and the classic four-step transport model. It uses the econometric method to link port throughput (that is, the aggregate demand for the local freight market) to economic variables and transforms the uncertainty in the economy of the port hinterland into a change in port throughput. Subsequently, it simulates the dynamic interactions among shippers, road freight companies, and railway competitors within a classic four-step transport framework. Thus, this framework provides a more in-depth understanding of the dynamic interplay between freight transport systems and the macroeconomic environment. Second, the system dynamics framework presents a more nuanced analysis of the economic and environmental impacts of ETTs. The causal interlinkages between the adoption of ETTs and the freight market are considered, and the impact of ETTs on the cost structure, transport efficiency, and pricing of road freight enterprises is explored. In this manner, the impact of the adoption of ETTs on the profit and GHG emissions of road freight enterprises is investigated. Considering the trade-off between economic performance and environmental performance, implications regarding the adoption of ETTs and the sustainable development of road freight companies are provided.

The remainder of this paper is framed as follows. Section 2 presents a literature review. The interaction among freight transport stakeholders is analyzed, and features of ETTs in the application to road transport are specified. Section 3 conceptualizes the system dynamics framework and constructs scenarios regarding the adoption of ETTs. Section 4 explores the impact of both macroeconomic uncertainty and the adoption of ETTs on road freight profit and GHG emissions. Section 5 compares the results between this study and existing studies and presents implications for the sustainable development of road freight enterprises. Section 6 summarizes the conclusions and suggests directions for future work.

## 2. Literature Review

### 2.1. Factors Influencing the Economic and Environmental Performance of Road Freight Enterprises

Road freight enterprises affiliated with ports are responsible for the transport of goods between ports and inland destinations. They play an essential role in all port-related logistic chains [14]. The economic and environmental performances of road freight enterprises are related to multiple factors, such as shippers’ mode choices, competition among different transport modes, strategic planning of port authorities, and the macroeconomy of the port hinterland [15]. Specifically, Melkonyan et al. [16] highlighted the driving effect of shippers’ mode preference on the changes in freight flow and carrier traffic flow. Kaack et al. [17] found that, due to the decline in competitor development, road freight experienced an evident growth as freight demand shifted from rail to road. Axsen et al. [18] scrutinized the response of freight systems to climate change policies (including price mechanisms, fuel switching regulations, etc.) and found that transport efficiency, general social welfare, and stakeholder perceptions are affected by these policies.

In addition to the factors related to freight transport, macroeconomic and social changes also influence the economic and environmental performance of road freight enterprises. Regarding the case of the present study, Shandong Province implemented *The Kinetic Conversion from Old to New Industries* [13]. This major long-term industrial upgrade planning has had a profound impact on freight types and volumes [19]. On the other hand, the recent COVID-19 pandemic has caused mobile restrictions and disruptions to the transportation network, introducing uncertainty for the road freight business. Regarding the freight market, the road freight industry in China has featured a narrow and low-concentration status. Most road freight companies are small, and the freight market is scattered and chaotic [20]. In recent years, the road freight industry in China has been undergoing a consolidation of freight capacity. With a more standardized fleet and an increased fleet scale, the vehicle utilization and pricing capability of road freight enterprises can be improved. The low-carbon performance of freight enterprises depends increasingly on management decisions, rendering economic efficiency critical [21]. ETTs have the potential to increase transport efficiency while reducing negative environmental impacts. A more standardized and scaled truck fleet is conducive to a smoother implementation and adoption of ETTs.

### 2.2. ETTs and Their Potential to Increase Transport Efficiency

In recent years, road freight enterprises in China have been under the pressure of both growth in economic profits and mitigation of climate change. Multiple scholars have explored feasible solutions for improving the operational efficiency and profitability of road freight enterprises [20,22,23]. They found that road freight enterprises in China are currently in a state of inefficient operational patterns and low technology deployment. In view of the economic growth in the port hinterland and the increase in port throughput, road freight enterprises affiliated with ports need to adapt to new economic conditions and stricter environmental regulations. The adoption of ETTs exhibits the potential to form a more efficient freight transport system [7]. This study includes the following ETTs.

- Eco-driving. Road freight enterprises retrofit freight vehicles with fuel-efficiency improvement systems to instruct alternative fuel-efficient driving behaviors. Barth and Boriboonsomsin [6] applied simulations and field experiments to explore the effect of eco-driving. They found that the fuel consumption of trucks decreases by 10–20% with eco-driving.
- Fleet platooning. Employing intelligent vehicle communication and automation technologies achieves shorter distances between trucks on highways [24]. Thus, fuel is saved by reducing resistance. Wadud et al. [7] reported a 3–25% reduction in fuel consumption by employing truck platooning.
- Vehicle utilization. This ETT refers to increasing the load rate of trucks and reducing empty vehicles. This involves the utilization of information technologies to form a vertical collaborative relationship between shippers and road freight enterprises [8]. As a result, the utilization rates of vehicles can be increased by sharing freight information. Mulholland et al. [1] reported an average of a 7.3–12.8% increase in the vehicle utilization rate by vertical collaborations between shippers and road freight enterprises.
- Optimized vehicle design. Via optimized vehicle design (adoption of pneumatics, improved tire design, and lightweight vehicle bodies) and transmission system improvements, energy use intensity during freight transport is reduced [9]. Delgado et al. [25] reported a 20–33% reduction in the energy use intensity for heavy-duty trucks in China by 2035.
- Renewable energy trucks. Chu and Majumdar [10] summarized the merits, challenges, and progress of alternative fuels in the transportation sector. In response to the need for a higher transport mileage and faster fuel refilling, Shandong Province emphasized the cultivation and application of hydrogen energy for heavy-duty trucks in *The Kinetic Conversion from Old to New Industries* initiative [13].

### 2.3. Research Gap

In summary, the mechanism of ETTs in increasing transport efficiency and reducing negative environmental impacts has been investigated in existing studies through truck experiments [26] or simulations and interviews [27]. The adoption of ETTs takes place in the freight market and changes freight economic and environmental performance. Modeling a market-wide context enables a more in-depth evaluation of the potential impact of ETT adoption. This study extends the analysis of ETTs’ impacts by including freight market factors such as mode choice, fleet evolution, optimal pricing, and market competition. Thus, a holistic perspective for the analysis of the impact of ETTs is established.

In addition, there is a lack of understanding of the costs and benefits in the application of these ETTs. Particularly under an uncertain macroeconomic environment, the impact of ETTs on road freight profit and GHG emissions has not been investigated. As a traditional energy-intensive industry, road transport needs to adapt to stricter environmental regulations while maintaining a reasonable level of profit. Exploring both the economic and the environmental impacts of ETTs is meaningful to the sustainable development of road freight enterprises.

## 3. Methodology

### 3.1. Theoretical Basis

**Figure 1:**
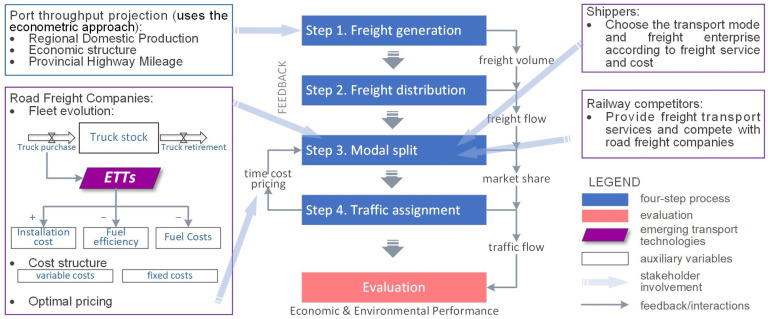
Theoretical framework of this study.

This study aims to explore the holistic impact of both the economic uncertainty in the port hinterland and the adoption of ETTs on the profitability and GHG reduction in road freight enterprises. Four types of stakeholders are involved in freight transport related to ports: shippers, road freight enterprises, railway competitors, and government authorities. We establish an analytical framework based on a view of stakeholders’ interaction. The approach for establishing the framework is system dynamics, which is particularly suitable for problems with continuous and dynamic quantities interconnected in feedback loops and circular causality [28]. The interaction among these stakeholders is simulated within the classic four-step transport theory [29,30]. The theoretical framework is illustrated in Figure 1.

### 3.2. System Dynamics Model

**Figure 2:**
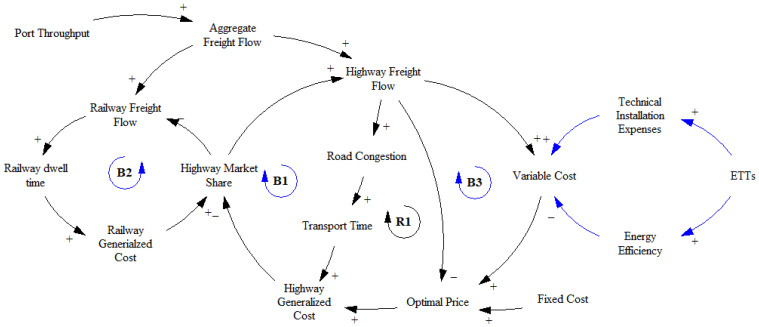
Causal loop diagram of the proposed system dynamics framework.

The theoretical framework is conceptualized in a qualitative causal loop diagram, which illustrates the interactive relationships among the main factors within the framework. The economic and environmental performances of road freight enterprises are influenced by three balancing loops and one reinforcing loop, as shown in Figure 2.

Balancing loop B1 illustrates that the growth in road freight volume tends to increase road congestion and, in turn, limits the increase in road market shares. Hu et al. [31] explained that, in regions and cities where road congestion is evident, rail transport and urban underground freight systems are being developed. The influence of road congestion on the road market share is reflected by the increased transport time and road generalized cost (including transport operating cost and time cost). Balancing loop B2 demonstrates a similar relationship among freight volume, congestion, dwelling time, and market share of the competitor, that is, railway transport. Reinforcing loop R1 and balancing loop B3 reflect the relationship between transport cost and freight pricing. The adoption of ETTs incurs additional installation costs for road freight companies. In turn, road freight companies may raise freight prices to shippers to offset this upfront deployment cost. This further changes road market shares.

The causal loop diagram is transformed into a quantitative stock–flow model, which is implemented using the Vensim DSS^®^ software package (Ventana Systems, Inc., Harvard, MA, USA). According to the classic four-step transport theory, the stock–flow model consists of four inter-related parts, which are specified as follows. Details of the model implementation are available in Section S1.

1. Freight generation. Ore, coal, liquid bulk, and containers in Qingdao port correspond to the demand for materials of production and the export of manufacturing by hinterland industries. Therefore, port throughput (which serves as shippers’ aggregate freight demand) is related to the economic development of the port hinterland. This study uses the econometric approach to convert hinterland economic trends into the estimation of aggregate port throughput [1,19,32]. Furthermore, the proposed framework takes into account the uncertainty of economic development by including the gross regional product and industrial structure as the key influencing variables for port throughput. The fitted correlations and the estimated long-term economic trends (addressed as economic scenarios) are combined to create the long-term projection of port throughput. The econometric model is expressed as Equation (1).

(1) $$pt_{t}=\beta_{0}+\beta_{1}gdp_{t}+\beta_{2}est_{t}+\beta_{3}Road_{t}+\varepsilon_{t}$$

Here, pt denotes port throughput. gdp, est, and Road denote regional domestic production, economic structure, and highway mileage, respectively. Coefficients fitted to these factors are denoted as $\beta_{i}$. ε is the residual term.

2. Freight distribution. Port throughput is the aggregated freight demand of the local freight market. It is converted into freight flows fl (in ton-kilometers) between port and inland destinations by multiplying the port throughput and the weighted transport distances L [12].

(2) $$fl_{t}=pt_{t}\times L$$

3. Mode split. The aggregate freight flow is assigned to different transport modes according to the market shares of the transport modes. Modal freight flows determine the business of freight enterprises, while mode shares depend on shippers’ mode choices. Railway transport is more efficient (with lower transport cost), which is conducive to reducing negative environmental impacts, but its greater waiting time leads to a lower preference of shippers [33]. Mode shares are estimated by the logit or the multinomial logit (MNL) models [30]. The random utility approach is used to synthesize the attributes related to mode choice (such as freight rate and transport time) into utility attributes to determine mode shares [34], and is expressed as Equation (3).

(3) $$\left\{ \begin{array}{l} RFMS_{t}=\frac{\exp(\alpha \times g_{t-1}^{})}{\sum_{k\in K}\exp(\alpha \times g_{t-1}^{})}\\ fv_{t}=fl_{t}\times RFMS_{t} \end{array}\right.$$

where both freight flow fl and market share RFMS determine the volume of freight operations for road freight companies (fv). RFMS is determined by the general cost g (determined by freight price and value of time).

4. Traffic assignment. Both the traffic volume and the capacity of the road network determine road transport time and fuel consumption. With an increase in traffic flow, congestion occurs more frequently [35]. The extended freight time and elevated costs feed back to the shippers, affecting the competitiveness of road freight enterprises. This study utilizes the speed-flow function (Equation (4)) to estimate the road transport time. The values of the parameters refer to Liu et al. [19].


(4) $$T_{t}^{RD}=\frac{1+e^{\mu +\gamma \times {\left(v_{t}/q_{t}\right)}^{3}\times \ln(v_{t}/q_{t})}}{\lambda \times S_{t}}$$

The impact of the adoption of ETTs is explored within the above four-step transport model. Road freight enterprises own trucks and provide transport services for shippers. They set the optimal freight pricing to obtain profits. Based on the cost structure proposed by Bösch et al. [36], this study adopts the classical optimal pricing theory to determine freight pricing [37]. The adoption of ETTs is related to fleet evolution, which is simulated and expressed in Equation (5).

(5) $$\left\{ \begin{array}{l} TS_{t}=\sum_{T\leq t-1}\sum_{x}\left(Purchase_{x,t}\times Survival_{x,t}\right)\\ Purchase_{x,t}=(DT_{t}-TS_{t})\times MS_{x} \end{array}\right.$$

where $TS_{t}$ denotes the stock of trucks at the end of year t. $Purchase_{x,t}$ and $Survival_{x,t}$ are the vehicle acquisition and vehicle survival rates, respectively. x is the fuel type of trucks. DT and MS represent truck demand and market share, respectively, for year t.

Based on the simulation of the truck fleet evolution, the freight cost and optimal pricing are derived in Equation (6).

(6) $$\left\{ \begin{array}{l} O(CI_{m})=\sum_{m}CI_{m}\times uc_{m}+M\\ p_{t}=\frac{p_{t-1}\times (e_{P}-1)}{2\times e_{P}}+\frac{O(CI_{m})}{2\times fv_{t}^{k}\times (1-r)} \end{array}\right.$$

where O represents the transport operating cost. CI refers to the mth operating cost item, and uc represents the unit costs corresponding to these cost items. M is the management cost. $p_{t}$ denotes the freight rate for the current period and is derived from the optimal pricing theory [37]. $e_{P}$ is the price elasticity. r denotes the value-added tax rate. The adoption of ETTs changes the fuel efficiency, cost structure, and market pricing of road freight companies. The freight market subsequently responds to such changes and ultimately leads to changes in the economic and environmental performance of road freight companies.

Finally, road freight profit and GHG emissions are estimated using Equations (7) and (8).

(7) $$PROFIT_{t}=fv_{t}\times p_{t}-O(CI_{m})$$

(8) $$GHG_{t}=\sum_{x}fv\times RFMS\times EI_{x}\times factor_{x}$$

where $PROFIT_{t}$ denotes road freight profit, which is derived by subtracting revenue by cost. $GHG_{t}$ represents road freight GHG emissions. $EI_{x}$ and $factor_{x}$ denote energy intensity and emission factors, respectively.

### 3.3. Input Parameters

Most of the exogenous parameters of the system dynamics framework were informed by the realistic status of the real system. The values of these exogenous parameters were obtained by either official statistics or onsite surveys or interviews. For example, the throughput of Qingdao port was extrapolated from the historical trend of economic development using econometric models. Data on port throughput were collected from the Wind Database [38], and the data related to explanatory variables, such as regional GDP, economic structure, and provincial road mileage, were obtained from official statistics [39]. The values of the parameters regarding freight transport activities (e.g., the value for average truck loading rate, average mileage, freight rate, and transport costs including vehicle procurement, maintenance, staff salaries, fuel prices, and road charges) were derived from interviews or onsite surveys. Experts and practitioners from Qingdao Port Group and multiple subsidiary companies of this group were interviewed. These subsidiary companies include road freight companies (e.g., Qingdao Evergreen Container Storage and Transport Co., Ltd., Qingdao, China), logistics companies (e.g., Qingdao Bonded Logistics Park), and freight forwarders (e.g., Shandong Ririshun Freight Forwarder Co., Ltd., Qingdao, China). The business of these subsidiary companies covers multiple aspects related to the hinterland transport of Qingdao port. Since most of the exogenous parameters have actual meanings, interviewees were asked to introduce relevant transport processes and to provide values regarding these parameters based on the data they collected or their expertise. These exogenous parameters and their values are summarized in Table 1.

### 3.4. Scenarios Regarding ETTs

Road freight enterprises affiliated with ports operate in an uncertain economic environment. The dynamic nature of the economic environment complicates the investigation of the impact of ETTs. Regarding the case of this study, *The Kinetic Conversion from Old to New Industries* initiative implemented in Shandong Province has a profound impact on the throughput of Qingdao port. This uncertainty in economic development is reflected by different predefined economic scenarios. The baseline scenario assumes a 5.5% growth rate in the macroeconomy of Shandong Province, according to the trend during the last decade. The high scenario assumes that the industrial upgrade will succeed and sets a 6% growth rate according to the goals set by the initiative [13].

The adoption of different ETTs influences the operation of road freight enterprises from different aspects. On the one hand, it exhibits potential for fuel savings and GHG reduction in road transport. On the other hand, the adoption of ETTs increases enterprise operation costs. Road freight enterprises compensate for the increase in operation cost by raising freight rates to shippers, which eventually affects shippers’ mode choices. This study considers five ETTs: eco-driving, fleet platooning, vehicle utilization, optimized vehicle design, and renewable energy trucks. The parameters corresponding to the application of these ETTs are listed in Table 2. The effects of these ETTs in increasing operation costs and reducing GHG emissions are derived from the data reported in the literature (Section 2.2). ETTs are deployed as the truck fleet evolves. It is assumed that the proportion of vehicles retrofitted with ETTs will grow linearly to 100% by 2035.

## 4. Results

### 4.1. Model Validation

**Figure 3:**
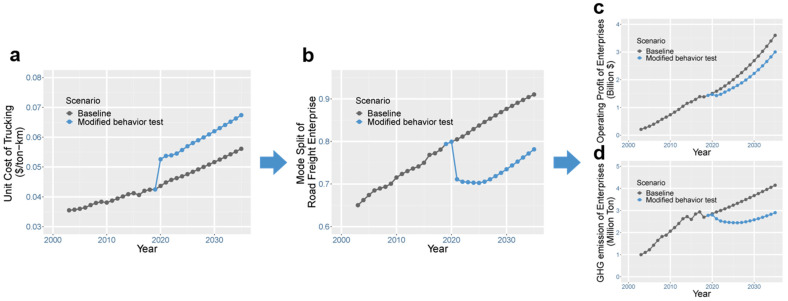
Modified-behavior test of the system dynamics model, in which (**a**) freight cost changes lead to changes in (**b**) market share, (**c**) enterprise profit, and (**d**) GHG emissions.

**Figure 4:**
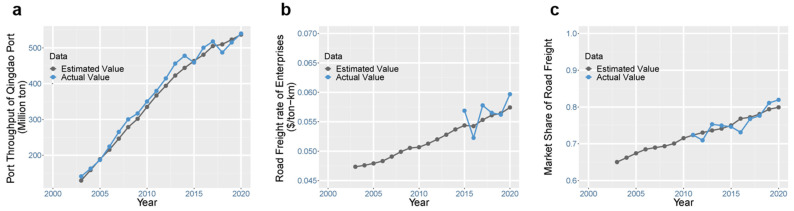
Behavior pattern test of the system dynamics model. The trends of the simulated value are compared with the actual historical data for (**a**) port throughput, (**b**) freight rate, and (**c**) road market share.

The proposed system dynamics framework needs to be validated before it is used to project the long-term impact of ETTs. The framework was tested by applying the classic system dynamics modeling validation procedure [43], which includes three categories of tests. (i) The structure confirmation test examines the theoretical basis of the model. The framework proposed in this study was developed based on the classic four-step transport theory (Section 3.1). The interaction of stakeholders is also coherent with the real behavior. This category of tests also includes the dimensional consistency test and the boundary adequacy test, whose results are presented and explained in Section S2. (ii) The modified-behavior test examines whether the simulated behavior is consistent with the real behavior when the values of some parameters are changed. The results of the modified-behavior test are shown in Figure 3. We set a 20% increase in road transport cost (Figure 3a) and checked whether the model captured stakeholders’ behavior. The results show that competitors (railway transport) acquire more market shares (that is, a reduction in road share, as shown in Figure 3b), which ultimately reduces road freight profit (Figure 3c) and GHG emissions (Figure 3d). This result suggests that the model captures stakeholder interactions as well as the behavior feedback effect. (iii) The behavior pattern test measures how accurately the model can reproduce the major behavior patterns exhibited by the real system. The simulated values of port throughput, freight rate, and market share were compared with the actual historical data. The results in Figure 4 show that the simulated value and the actual value are well correlated in their trends. This validation provides confidence in the structure of the proposed system dynamics framework.

### 4.2. Impact of Macroeconomic Uncertainty on Road Freight Profit and GHG Emissions

**Figure 5:**
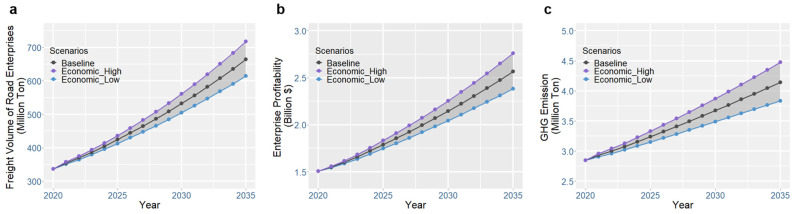
Impact of macroeconomic uncertainty on (**a**) freight volume, (**b**) profit, and (**c**) GHG emissions of road freight enterprises.

As the main economic hinterland of Qingdao port, Shandong Province is undergoing a major industrial upgrade. Road freight profit and GHG emissions are determined by road freight volume, which depends on two factors: macroeconomic trends (which determine the total freight volume) and freight market competition (which determines the market share of the road). The impacts of macroeconomic uncertainty on road freight volume, profit, and GHG emissions are illustrated in Figure 5.

The results show that, in the baseline scenario, which assumes an increase of 123% from 2020 to 2035, the throughput of Qingdao port (serving as the aggregate freight demand for the local freight market) will increase by 64%. As a result, the profit of road freight enterprises will grow by 78% (to USD 2.57 billion, as shown in Figure 5b) due to the increase in freight volume (Figure 5a), in which 14% of the gains are attributed to the increase in road freight market shares. With the increase in freight demand, GHG emissions will grow by 45% due to the increase in road freight activities (Figure 5c). The uncertainty in macroeconomic growth is addressed by constructing an economic high scenario and an economic low scenario. These scenarios correspond to economic growth rates of between 5% and 6%. These growth rates are set referring to Sun et al.’s [44] extrapolation of China’s economic development until 2035. The results show that, regarding different economic scenarios, the trends for road freight profit (Figure 5b) and GHG emissions (Figure 5c) are the same. This indicates that, in the absence of ETTs, the profitability of road freight conflicts with the goal of climate change mitigation.

### 4.3. Impact of ETTs on Road Freight Profit and GHG Emissions

**Figure 6:**
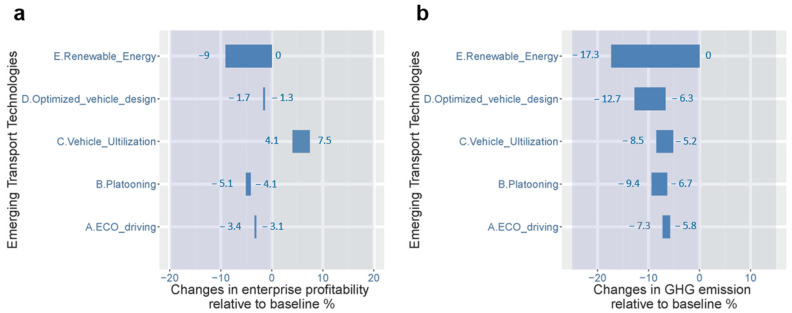
Impact of ETTs on (**a**) profit and (**b**) GHG emissions of road freight. The impact is illustrated in terms of percentage changes in profit and GHG emissions until 2035 compared to the value of the baseline scenario.

In view of the conflict between road freight profitability and climate change mitigation (particularly in an increasing macroeconomic development trend), introducing ETTs to road freight is necessary. We consider five candidate ETTs for road freight. The specifications of these five ETTs are explained in Section 2.2, and parameters corresponding to the adoption of ETTs are summarized in Table 2. The impact of ETTs on road freight profit and GHG emissions (in terms of the percentage in the change in profit and GHG emissions compared to the value of the baseline scenario) is illustrated in Figure 6.

Figure 6a shows that the adoption of most of the candidate ETTs (including eco-driving, fleet platooning, optimized vehicle design, and renewable energy trucks) leads to a decline in road freight profit. This is due to a higher cost for the installation or retrofitting of truck devices or the use of renewable fuels for applying these ETTs, even though these ETTs increase fuel efficiency and reduce transport operating costs. In contrast, increasing vehicle utilization (via vertical collaboration of freight markets) is profitable due to cost savings in vehicle acquisition and road tolling. Although some intangible costs for the collaboration of freight markets are not included, increasing vehicle utilization is still more beneficial than other ETTs because it enhances the effect of scale economies of transport.

Figure 6b shows that all of the candidate ETTs exhibit a positive effect in reducing GHG emissions from road freight. The effect of ETTs on road freight GHG reduction depends on both the adoption of these ETTs in road freight and the competition from railway transport (potential freight shift from road to railway); that is, ETTs reduce fuel consumption from road transport activities, but they incur greater road freight rates, which lower shippers’ preference for road transport and shift freight from road to railway. Thus, the estimated GHG reduction percentages in Figure 6b may exceed the values reported in previous studies (Mulholland et al., 2018). Another notable feature in Figure 6 is that the effect of ETTs on GHG reduction (Figure 6b) is generally proportional to the cost of applying these ETTs (reduction in profit in Figure 6a). This feature suggests that introducing a carbon-based compensation mechanism may be conducive to the adoption of ETTs.

**Figure 7:**
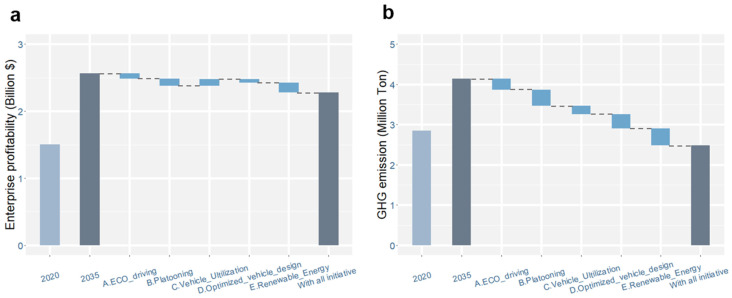
Effects of ETTs on (**a**) profit and (**b**) GHG emissions of road freight by 2035 when ETTs are deployed concurrently. The grey bars on the left are the initial values in 2020. The dark grey bars (the second and eighth bars) show the projected values for 2035 without and with the implementation of all ETTs, and the short blue bars show the impact of ETTs.

Next, we further explore the effects of ETTs on road freight profit and GHG emissions when these ETTs are implemented concurrently. The results are shown in Figure 7.

In Figure 7, the left two columns in each chart represent road freight profit/GHG emissions in 2020 and 2035 in the baseline scenario (in which ETTs are not implemented). The columns on the right show the effects of ETTs on profit/GHG emissions by 2035. The results show that, with the adoption of all five ETTs, the profit of road freight enterprises by 2035 (the first right column) declines by 12% (compared with the value of the baseline scenario). GHG emissions are reduced more evidently, with a 40% emission reduction by 2035.

### 4.4. Prioritization of ETTs Based on Sensitivity Analysis

**Figure 8:**
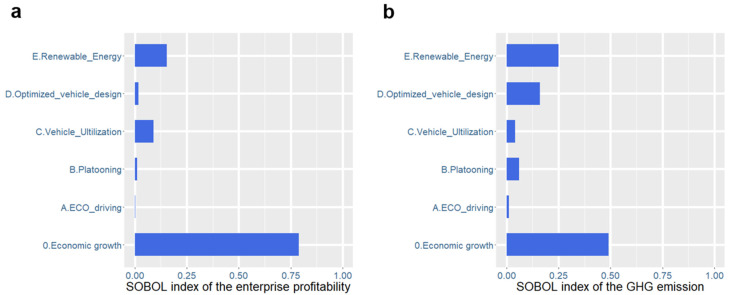
Sensitivity analysis of ETTs’ impact on (**a**) road freight profit and (**b**) GHG emissions.

Figure 7 shows a substantial reduction in GHG emissions when all of the candidate ETTs are implemented. However, adopting ETTs incurs additional costs to road freight enterprises, whose budgets are usually limited [45]. Thus, prioritizing the candidate ETTs is meaningful to road freight enterprises. A sensitivity analysis of the impacts on profit and GHG emissions was performed. This was executed with the Sobol index method [46], which is commonly used for global sensitivity analyses. Working within a probabilistic framework, Sobol sensitivity analysis decomposes the variance of the output of the model or system into fractions that can be attributed to inputs or sets of inputs. We sampled the value space of the exogenous parameters in Table 2 to generate the Sobol sample. The data generated were then used to calculate the sensitivity to input parameter changes on road freight profit and GHG emissions. Sobol sensitivity analysis was performed using the SALib package in Python (available at https://github.com/SALib/SALib (accessed on 10 October 2021)). The results are shown in Figure 8.

**Figure 9:**
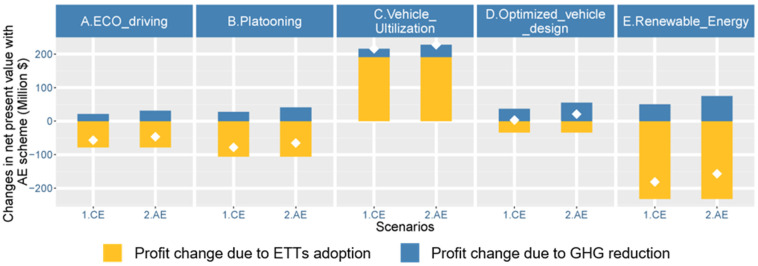
Changes in the net present value with the continued effort (CE) scheme and the accelerated effort (AE) scheme.

The results show that both road freight profit and GHG emissions are most sensitive to the macroeconomic environment. This is because economic growth leads to an increase in freight demand, which directly determines the amount of road freight activities as well as road freight profit and GHG emissions. Figure 8 also shows that GHG reductions are sensitive to the adoption of ETTs. Thus, introducing a carbon-based compensation mechanism is necessary to encourage road freight enterprises to accept and apply these ETTs [47]. Two carbon trading schemes are introduced: the continued effort (CE) scheme (USD 35/tCO_2_) and the accelerated effort (AE) scheme (USD 52/tCO_2_) [48]. Via the carbon trading schemes, reductions in GHG emissions are converted into profit. Both the profit loss from the adoption of ETTs and profit gains via emission reduction are summarized in the net present value. The results are shown in Figure 9.

In Figure 9, the yellow parts and the blues part indicate the profit loss and gains due to the adoption of ETTs, and the white point is the net present value. The results show that vehicle utilization takes the first priority because it reduces both transport operating costs and GHG emissions by enhancing scale economies of transport. Optimized vehicle design ranks second in terms of net present value. Its profit by GHG reduction (with the carbon trading price) generally compensates for the additional application cost. Eco-driving ranks third, but its net present value is negative, as are those of the other two ETTs (fleet platooning and renewable energy trucks). It is noteworthy that this result depends on the predefined carbon trading price. Thus, ETTs with negative net present values may be more feasible over a longer time horizon when the carbon trading price increases [49]. In addition, greater investments in research and development for these ETTs are conducive to reducing application costs and increasing the energy-saving performance of ETTs.

## 5. Discussion

Existing studies have investigated the fuel-saving potential of ETTs via vehicle experiments. This study extends the analysis from a freight-market-wide context. Introducing technology penetration to the simulation of truck fleet evolution, the impact of ETTs on reducing GHG emissions from the entire road freight industry is explored. For some of the ETTs, the results of this study are close to the values reported in existing studies. For example, the GHG reduction potential of vehicle utilization in this study is close to the results of Wong et al. [50] because both studies share similar assumptions. Mulholland et al. [1] explored the comprehensive impact of ETTs on global road freight emission reductions. Their results indicate a 40–45% reduction in freight energy intensity in China in 2035, which is in line with the optimistic scenario presented in this study (Figure 7b). On the other hand, the GHG reduction potential of eco-driving in this study is lower than the value reported by Sullman et al. [51] because this study considers a long-term technology penetration process.

The system dynamics assessment framework integrates econometric estimation and freight transport simulation. Thus, it provides a holistic view of the impact of ETTs on road freight profit and GHG emissions under a changing macroeconomic environment. Regarding the hinterland transport of Qingdao port, policy implications are provided as follows.

- In a growing economy, it is difficult to coordinate the demand for freight delivery and the goal of GHG reduction. Economic growth in Shandong Province will lead to increased freight demand in Qingdao port. The environmental issues arising from freight traffic are a major challenge for road freight companies and port authorities. In this circumstance, the adoption of ETTs is an effective means to satisfy freight demands while mitigating negative environmental impacts.
- ETTs are conducive to improving transport efficiency and reducing operational costs. However, their adoption cannot fully offset the cost of equipment installation. Thus, economic incentives are necessary to raise freight companies’ intention to adopt ETTs. General policy levers include subsidies or carbon trading schemes.
- Regarding the case of this study, optimizing vehicle design and improving the efficiency of vehicle use are currently plausible options. In contrast, the adoption of eco-driving and fleet platooning is either too costly or only being piloted. Nevertheless, companies can partially realize the benefits of both options through driver training [51].

## 6. Conclusions

ETTs have the potential to increase fuel efficiency and reduce GHG emissions from freight transport. A system dynamics analytical framework is established to explore the impact of ETTs on the profitability and GHG emissions of road freight enterprises affiliated with ports, especially under the uncertainty in macroeconomic development trends. The proposed framework simulates a series of transport activities that are related to the operation of freight enterprises, such as freight demand, mode choice, truck fleet evolution, cost structure, and pricing mechanism. Therefore, the framework is able to estimate and compare the impact of different ETTs on the economic and environmental performance of freight enterprises. In this study, road freight enterprises affiliated with Qingdao port in China are taken as a case study to demonstrate the proposed analytical framework. Notable results are summarized as follows.

- With regional macroeconomic growth, the throughput of Qingdao port increases. This leads to an increase in the aggregate freight demand for local freight companies. As a result, the profit of the road freight enterprises affiliated with Qingdao port will reach USD 2.38–2.76 billion by 2035, which is an increase of 58–83% compared to the profit in 2020. Meanwhile, GHG emissions from road freight will increase to 3.8–4.5 million tons by 2035, an increase of 34.5–45.4% compared to the emissions in 2020 due to the growth in freight demand.
- All of the candidate ETTs exhibit a positive effect on reducing GHG emissions from road transport, but they also cause profit losses due to a high application cost, even though they reduce transport operating costs by providing fuel savings.
- Both road freight profit and GHG emissions are most sensitive to macroeconomic development because economic growth leads to an increase in freight demand and transport activities. GHG reductions are sensitive to the adoption of ETTs.
- A prioritization of the candidate ETTs is necessary for road freight enterprises, whose expenditure is usually limited; this entails the introduction of a carbon-based compensation mechanism. Vehicle utilization, optimized vehicle design, and eco-driving take the first three priorities during adoption by road freight enterprises. Other ETTs may be more feasible over a longer time horizon.

There are some limitations of the present study. First, this study assumes a linear penetration process of ETTs, which reaches full application within local road freight industry by 2035. In future work, a technology diffusion model can be introduced to simulate the ETT penetration process more accurately. Second, this study does not consider the long-term improvement in technology performance and the utilization of financial instruments. In view of a high cost in adopting ETTs, green financial instruments can be considered. Using the proposed framework, we can explore the implementation scheme of green financial instruments for a wider adoption of ETTs.

## Figures and Tables

**Table 1:** Exogenous parameters of the system dynamics model.

Parameter	Value	Unit	Source
Throughput of Qingdao Port in 2020	540	million ton	[38]
Hinterland macroeconomic data	with lookup	-	[39]
Average freight mileage	326	km	Average data weighted by regional economy
Average truck payload	35	ton	Onsite survey
Average annual mileage of heavy trucks	120,000	km	Onsite survey
Heavy truck stock in 2020	50,000	vehicle	Extrapolated
Purchased heavy trucks in 2020	9000	vehicle	Extrapolated
Vehicle acquisition costs	40,000	USD/vehicle	Onsite survey
Staff salaries	1400	USD/person/month	Onsite survey
Diesel price	1086	USD/ton	Onsite survey
Road charges	0.2	USD/km	Onsite survey
Shipper perceived time value	0.72	USD/ton/hour	Interview findings
Highest speed of trucks	80	km/hour	Onsite survey
Fuel consumption for road transport	18	tce/million tkm	Energy consumption [40]
GHG emission factor of Road	2.146	ton/tce	[41]
Freight rail speed	100	km/hour	Documents on railway construction standards
Average dwelling time for railway collections	2	day	Onsite survey

Notes: tkm denotes ton·kilometer. tce denotes tons of coal equivalent.

**Table 2:** Scenarios regarding macroeconomic uncertainty and the adoption of ETTs.

Scenario	Parameter Changed	Penetration	Parameterization			Cost	Source
			Baseline	Low	High		
Economic trends	economic growth	/	5%	5.5%	6%	/	[13]
Eco-driving	energy intensity	100%	0	10%	20%	USD 30,000	[6]
Fleet platooning	energy intensity	100%	0	3%	25%	USD 40,000	[7]
Vehicle utilization	freight activity	100%	0	7.3%	12.8%	/	[1]
Optimized vehicle design	energy intensity	/	2%	Baseline + 0.5%	Baseline + 1%	Cost-efficiency improvement curves [42]	[25]
Renewable Energy	energy mix	Hydrogen 10% LNG 10%	/	/	/	Hydrogen trucks: USD 240,000 LNG trucks USD 50,000	/

## Data Availability

Data is contained within the article or Supplementary Material.

## Supplementary Materials

The following supporting information can be downloaded at: https://www.mdpi.com/article/10.3390/ijerph192215077/s1, Section S1 presents the stock flow diagram and the main equations used in the system dynamics model. Section S2 shows the results of the dimensional consistency test and the boundary adequacy test of the system dynamics model [43,52].
