# Supplementary material for: Impact of Emerging Transport Technologies on Freight Economic and Environmental Performance: A System Dynamics View
Source: Int J Environ Res Public Health. 2022 Nov 16;19(22):15077. doi: 10.3390/ijerph192215077 (PMC9690938; doi:10.3390/ijerph192215077)
Supplement: Supplementary file 1 [file ijerph-19-15077-s001.zip › ijerph-1989975-supplementary.pdf]

Supplementary Materials

Impact of Emerging Transport Technologies on Freight Economic and Environmental Performance: A System Dynamics View

Taolei Guo, Junjie Chen and Pei Liu *

Department of Logistics Management, Business School, Shandong University, Weihai 264209, China
* Correspondence: liupei@sdu.edu.cn

Section S1. Implementation of the SD model in Vensim DSS®

This paper uses system dynamics model to project the future (2020-2035) economic and environmental performance of freight companies. The manuscript presents the framework and formulas of the model in detail. In the appendix, we describe the stock flow diagram of the model. Market share and freight activity were projected using the traditional four-step forecasting approach. The behavior of freight companies is then taken into account by modelling the truck fleet evolution, cost structure and optimal pricing.

S1-1. Freight generation

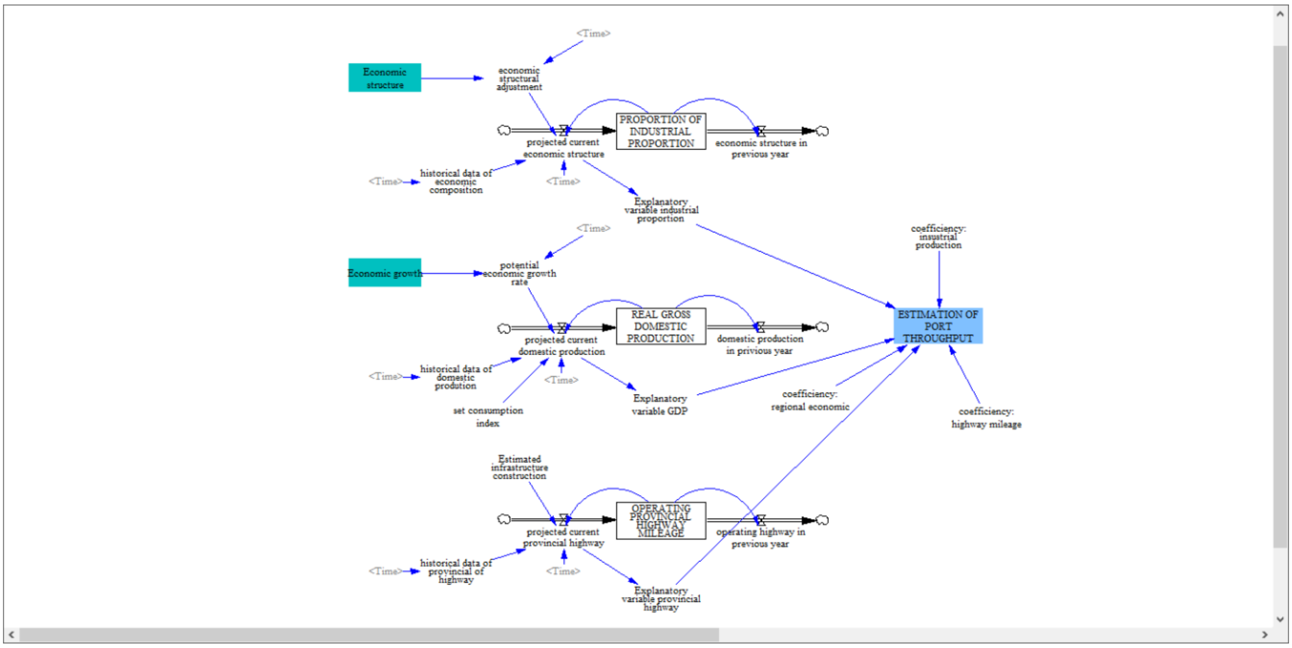

Note: Qingdao port throughput is projected using an econometric model, with explanatory variables being regional GDP, secondary industry percentage and provincial highway mileage.

Figure S1-1. Stock-flow diagram of freight generation

S/N	Equation	Explanation
1	"historical data of port throughput" = WITH LOOKUP (Time, ([(2000, 0)- (2017, 600)], (2000, 86.6071), (2001, 104.229), (2002, 122.516), (2003, 140.9), (2004, 162.65), (2005, 186.78), (2006, 224.15), (2007, 265.02), (2008, 300.29), (2009, 316.684), (2010, 350.12), (2011, 379.71), (2012, 414.648), (2013, 456.13), (2014, 477.82), (2015, 458.95), (2016, 500.36), (2017, 517.86)))<br>Units: Million TON	Historical values of Qingdao port throughput.
2	ESTIMATION OF PORT THROUGHPUT = -55.036 + "co-efficiency: regional economic" * Explanatory variable GDP + "co-efficiency: industrial production" * Explanatory variable industrial	Multivariate regression equation for predicting port throughput.

	proportion + "co-efficiency: highway mileage" * Explanatory variable provincial highway<br>Units: Million TON	
3	"Historical Data & Estimated Data" = IF THEN ELSE (Time<=2017, historical data of port throughput, ESTIMATION OF PORT THROUGHPUT)<br>Units: Million TON	The data before 2017 are input according to the real data obtained, and the subsequent data are projected by multivariable regression method.

S1-2. Freight distribution

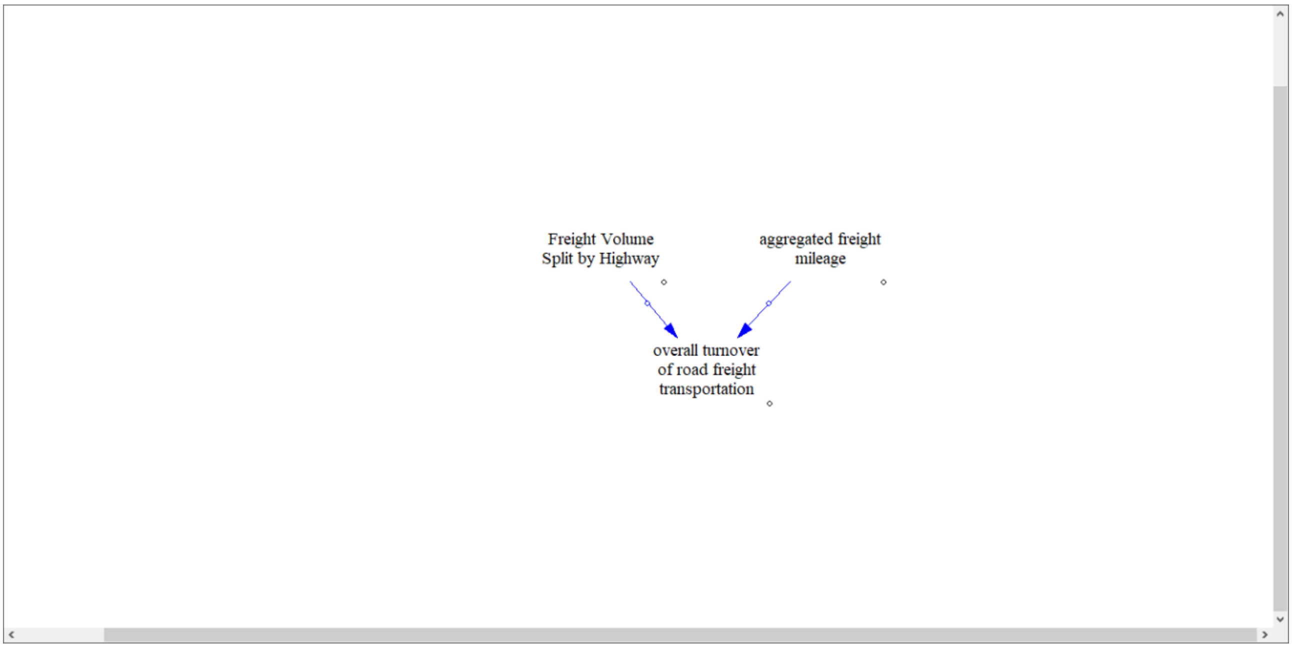

Note: The freight volume is converted into freight flows by multiplying the weighted transport mileage.

Figure S1-2. Stock-flow diagram of freight distribution

S/N	Equation	Explanation
1	overall turnover of road freight = aggregated freight mileage * Freight Volume Split by Highway<br>Units: Million TON*km	Traffic volume (turnover) of road freight

S1-3. Mode split

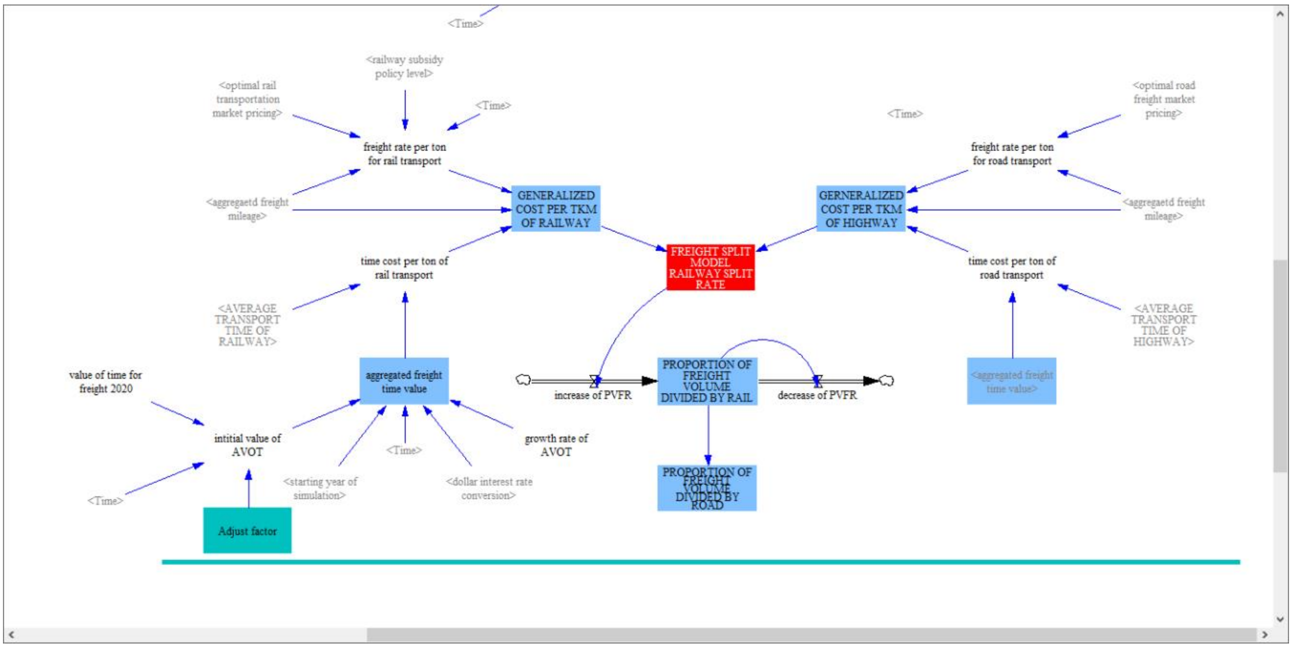

Note: The market share of freight companies is calculated based on the generalized costs of the different modes of freight transport.

Figure S1-3. Stock-flow diagram of mode split

S/N	Equation	Explanation
1	AVERAGE TRANSPORT TIME OF HIGHWAY = aggregated freight mileage*"time-consuming per kilometer of highway"<br>Units: hour/vehicle	Average time consumed by road and rail freight vehicles
2	AVERAGE TRANSPORT TIME OF RAILWAY = aggregated freight mileage/scheduled speed of the train + scheduled transport node wait time<br>Units: hour/vehicle	
3	GENERALIZED COST PER TKM OF RAILWAY = (freight rate per ton for rail transport + time cost per ton of rail transport) / aggregated freight mileage<br>Units: $/ton	Generalized cost includes trucking/railway operating cost and time cost.
4	GERNERALIZED COST PER TKM OF HIGHWAY = (freight rate per ton for road transport + time cost per ton of road transport) / aggregated freight mileage<br>Units: $/ton	
5	FREIGHT SPLIT MODEL RAILWAY SPLIT RATE = EXP (-Spread Parameter * Generalized Cost per TKM of Railway) / (EXP (-Spread Parameter * Generalized Cost per TKM of Railway) + EXP (- Spread Parameter * Generalized Cost per TKM of Highway))<br>Units: Dmnl	Logit model for freight split rate calculation.

S1-4. Traffic assignment

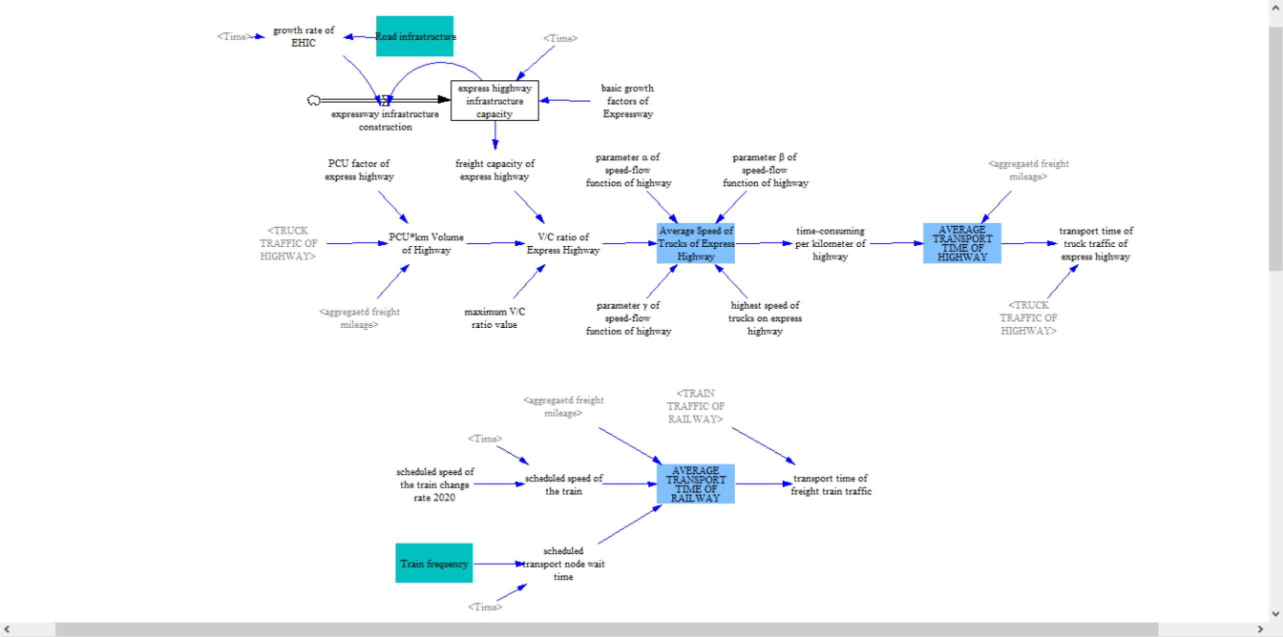

Note: The established flow rate relationships are used to calculate the timing of road freight, while the level of rail service is determined based on the liner's schedule.

Figure S1-4. Stock-flow diagram of traffic assignment

S/N	Equation	Explanation
1	express highway infrastructure capacity = IF THEN ELSE (Time<>2020, expressway infrastructure construction, expressway infrastructure construction + express highway infrastructure capacity * basic growth factors of Expressway)<br>Units: pcu*km/Year	Estimation of future road freight capacity growth.
2	"V/C ratio of Express Highway" = IF THEN ELSE ("PCU*km Volume of Highway" / freight capacity of express highway > "maximum V/C ratio value", "maximum V/C ratio value", "PCU*km Volume of Highway" / freight capacity of express highway)<br>Units: Dmnl	The ratio of highway traffic flow to design capacity can indicate the degree of highway congestion. It is generally considered that highways greater than 0.6 are congestion
3	Average Speed of Trucks of Express Highway = ("Parameter α of Speed - flow Function of Highway" * Highest Speed of Trucks on Express Highway) / (1 + "V/C ratio of Express Highway" ^ ("Parameter β of Speed - flow Function of Highway" + "Parameter γ of Speed - flow Function of Highway" * "V/C ratio of Express Highway" ^ 3))<br>Units: km/hour*vehicle	Relationship between average road traffic flow and freight speed

S1-5. Costs and pricing for freight companies

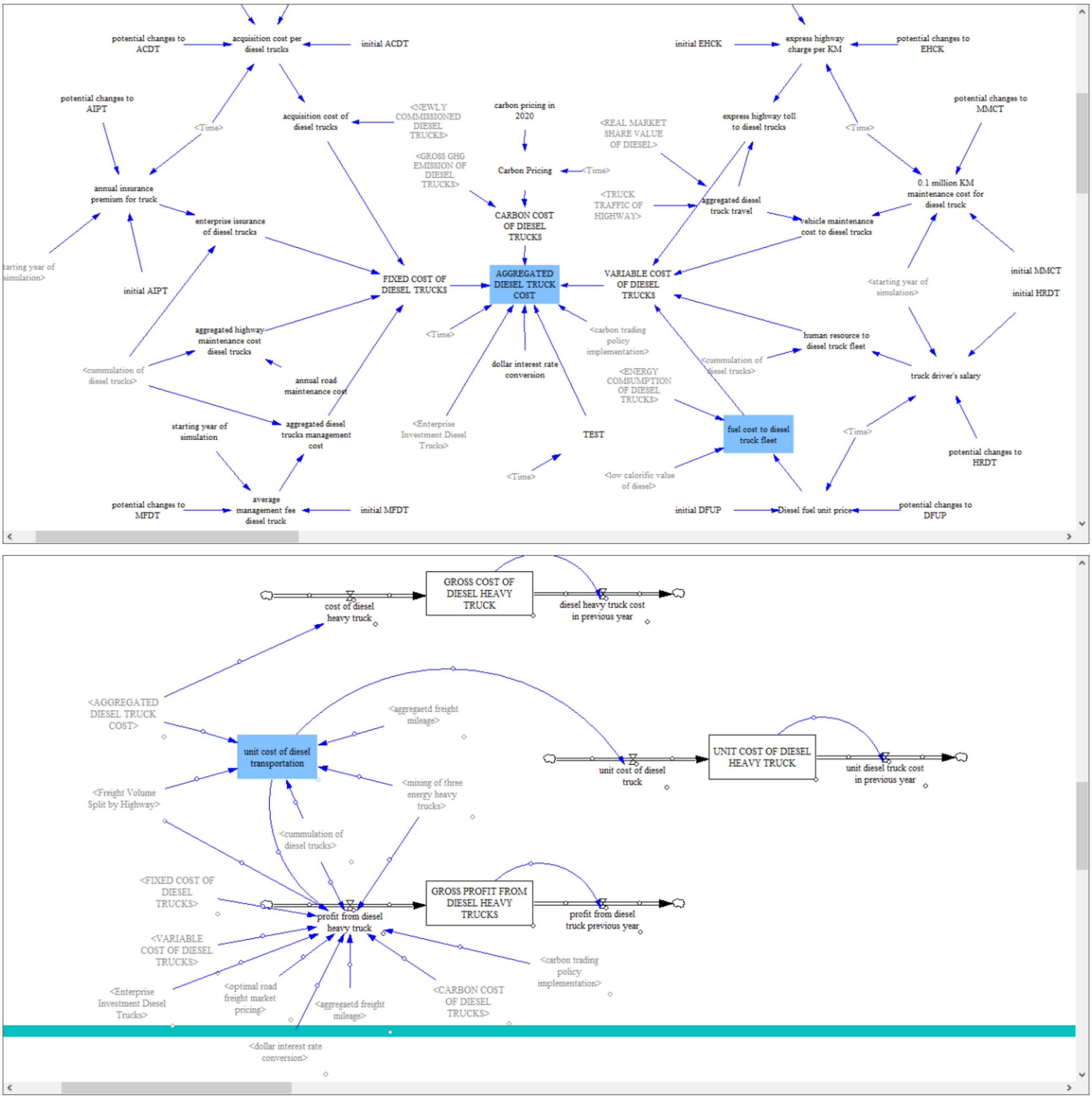

Figure S1-5. Stock-flow diagram of cost structure and optimal pricing for freight companies

S/N	Equation	Explanation
1	AGGREGATED DIESEL TRUCK COST = IF THEN ELSE (Time < 2020, (FIXED COST OF DIESEL TRUCKS + VARIABLE COST OF DIESEL TRUCKS) / dollar interest rate conversion, (FIXED COST OF DIESEL TRUCKS + VARIABLE COST OF DIESEL TRUCKS + CARBON COST OF DIESEL TRUCKS * carbon trading policy implementation) / dollar interest rate conversion) * TEST + Enterprise Investment Diesel Trucks<br>Units: $	Estimation of the overall cost of a freight company

2	FIXED COST OF DIESEL TRUCKS = acquisition cost of diesel trucks + aggregated diesel trucks management cost + aggregated highway maintenance cost diesel trucks +enterprise issuance of diesel trucks<br>Units: $	Cost structure
3	VARIABLE COST OF DIESEL TRUCKS = express highway toll to diesel trucks + fuel cost to diesel truck fleet + human resource to diesel truck fleet + vehicle maintenance cost to diesel trucks<br>Units: $	
4	optimal freight market pricing = weighted average freight cost / (2 * (1 - highway carrier business tax rate)) + ROAD TRANSPORTATION PRICING / 2 - ROAD TRANSPORT PRICING / (2 * (freight rate elasticity of road transportation))<br>Units: $/ton*km	Freight companies adjust pricing according to cost and price elasticity.
5	profit from diesel heavy truck = Freight Volume Split by Highway * (optimal road freight market pricing - unit cost of diesel transportation) * aggregated freight mileage<br>Units: $	Estimation of economic performance of freight transport enterprises
6	GROSS GHG EMISSION OF DIESEL TRUCKS = ENERGY COMSUMPTION OF DIESEL TRUCKS * emission factor for diesel transportation<br>Units: Million ton	Estimation of greenhouse gas emissions from freight transport companies

Section S2. Validation of the system dynamic model

The model is used to project the long-term economic and environmental performance for freight companies. In order to produce credible results, we conducted model validation by applying the classic system dynamics modeling validation procedure proposed by Barlas (1996). The manuscript presents the structure-confirmation test, the modified-behavior test and the behavior pattern test, which are important to validity of internal structure of the model. The appendix is supplemented with the Dimensional Consistency Test and the Boundary Adequacy test to illustrate the consistency of the model with the perception of reality.

Dimensional Consistency Test: The dimensional consistency test is performed using the unit test provided by the Vensim software. The results are presented in Fig. S2-1, which shows that the model passes the dimensional consistency test.

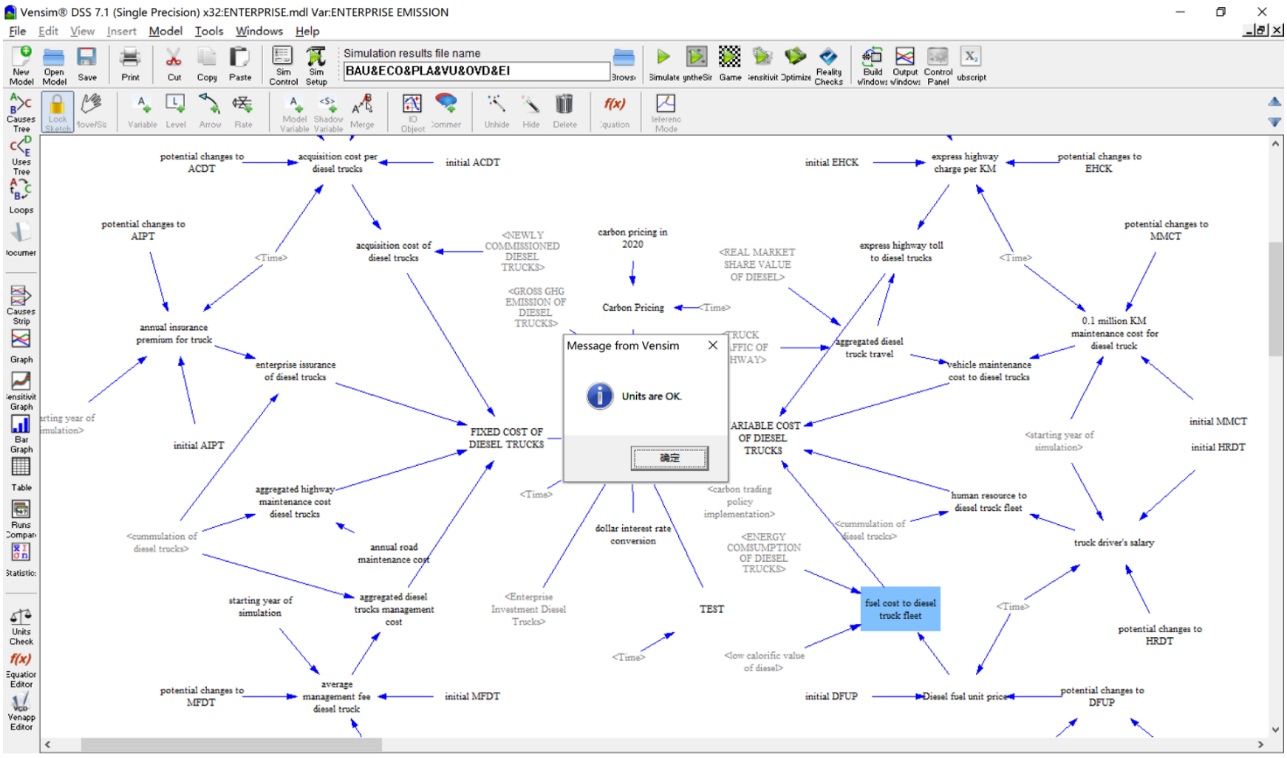

Figure S2-1. Results of Dimensional Consistency Test

Boundary Adequacy tests: The boundary adequacy test attempts to indicate whether the aggregation and the elements included in the model are adequate to provide insight into the objectives of the study (Schwaninger and Groesser, 2011). The model is used to evaluate the economic and environmental performance for freight companies when ETTs are adopted. Freight activities and dynamic interaction of stakeholders are two main aspects need to be represented in the model. The classic four-step transportation framework is capable of including freight activities into the model. On this basis, we take stakeholders and their interactive behavior into account, including shipper choice, fleet evolution, and competitor participation. In light of the extensive consideration of factors associated with the economic and environmental performance for freight transport, the boundary adequacy of the model is validated.

References

Barlas, Y. (1996) Formal aspects of model validity and validation in system dynamics. *System Dynamics Review* 12, 183-210.

Schwaninger, M., Groesser, S. (2011) System Dynamics Modeling: Validation for Quality Assurance. *Complex Systems in Finance and Econometrics* ed Meyers, R.A. Springer New York, New York, NY, pp. 767-781.
